# Cytarabine delivered by CD44 and bone targeting redox-sensitive liposomes for treatment of acute myelogenous leukemia

**DOI:** 10.1093/rb/rbac058

**Published:** 2022-08-24

**Authors:** Hao Wu, Yuan Gao, Jia Ma, Maosong Hu, Jing Xia, Shuting Bao, Yuxi Liu, Kai Feng

**Affiliations:** College of Materials & Chemical Engineering, Chuzhou University, Chuzhou 239000, China; Department of Oncology, Beijing Shunyi Hospital, Shunyi District, Beijing 101300, China; Department of Neurology, Beijing Shunyi Hospital, Shunyi District, Beijing 101300, China; College of Materials & Chemical Engineering, Chuzhou University, Chuzhou 239000, China; College of Materials & Chemical Engineering, Chuzhou University, Chuzhou 239000, China; College of Materials & Chemical Engineering, Chuzhou University, Chuzhou 239000, China; College of Materials & Chemical Engineering, Chuzhou University, Chuzhou 239000, China; Department of Neurology, Beijing Shunyi Hospital, Shunyi District, Beijing 101300, China

**Keywords:** cytarabine, liposomes, acute myeloid leukemia, hyaluronic acid

## Abstract

Acute myelogenous leukemia (AML) remains a serious fatal disease for the patients and effective treatment strategies are urgently needed. Based on the characteristics of the AML, we developed the CD44 and bone targeting liposomes delivery system decorated with the redox-cleavable polymer. First, ALN-HA was obtained by amination between alendronate (ALN) and hyaluronic acid (HA), and cholesterol (Chol) was coupled by a disulfide linker (-SS-) with biological reducibility to obtain the goal polymer, ALN-HA-SS-Chol, decorated the liposomes loaded with the Cytarabine (AraC). ALN-HA-SS-AraC-Lip exhibited a spherical morphology with the diameter of 117.5 nm and expanded at the environment of 10 mM dithiothreitol. Besides, compared with other groups, ALN-HA-SS-AraC-Lip showed benign hydroxyapatite affinity *in vitro* and bone targeting in C57/BL6 mice, also, ALN-HA-SS-AraC-Lip exhibited encouraging antitumor which significantly reduced the white blood cell amount in bone marrow and blood smear caused by AML model, besides, the dual targeting liposomes also prolong the survival time of mice. In conclusion, the bone and CD44 dual targeting liposomes with redox sensitivity could target to the leukemia stem cells regions and then uptake by the tumor cells, which would be a valuable target for the treatment of the AML.

## Graphical Abstract

**Graphical Abstract:**
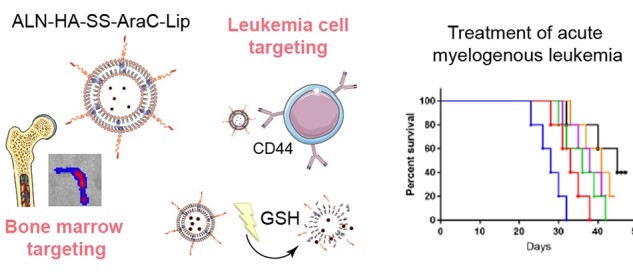

## Introduction

Acute myelogenous leukemia (AML) is a malignant disease, which is characterized by an abnormal proliferation and maturation stagnation of myeloid cells in the bone marrow, frequently resulting in hematopoietic insufficiency [1, 2]. At present, chemotherapy is almost the only method for the treatment of AML clinically. For decades, the standard induction for patients with AML has been the combination of cytarabine with anthracycline (daunorubicin or idarubicin). This standard regimen, called ‘7 + 3’, is a continuous cytarabine infusion for 7 days with chosen anthracycline given for the first 3 days [3]. After initial remission following the induction therapy, patients will undergo high doses of cytarabine therapy to eradicate residual leukemia; therefore, AraC is the main chemotherapeutic drug for the treatment of AML that prevents cancer cell growth by interfering with DNA synthesis. However, the utilization of AraC is limited due to its low stability after intravenous administration and the narrow therapeutic window [4]. Meanwhile, the hydrophilic character of AraC deeply limits the intracellular uptake because of the low membrane permeability of the molecule. The above issues raise the need for improved cellular uptake, stability and therapeutic effect, and provide new impetus for the present study.

To solve the above problems, nanoparticles including micelles and liposomes play a key role in this field. The construction of a nanoparticle is bond to introduce a lot of excipients compared with therapeutic agents. Although most of the excipients are non-toxic, nanoparticle drug delivery system is not safe. Therefore, improving the efficiency of drug treatment is key for nanoparticle drug delivery. Traditional chemotherapy for leukemia can only kill the differentiated cancer cells in the blood vessel and hardly affect the tumor stem cells in the bone marrow. In this study, the redox-sensitive liposomes targeting the bone and CD44 were developed for the treatment of AML. The functionalized liposomes not only had benign bone and CD44 dual targeting but also could release the drug quickly in the AML cell environment.

Alendronate (ALN) is a kind of bisphosphonates that exhibits a high affinity for the bone mineral hydroxyapatite (HAP), making it as a good candidate for the bone targeting agent to deliver drugs to the bone site [5, 6]. For example, Zhu *et al.* [7] designed ALN-functionalized polyethylene glycol (PEG) nanoparticles to deliver the antitumor drug bortezomib, and the *in vivo* biodistribution studies indicated that the fluorescence signal of the ALN-functionalized nanoparticles was ∼4 times higher in the bone tumor than that in the normal nanoparticles. Liu *et al.* [8] utilized ALN to develop the bone-targeting liposomes for the delivery of the anti-osteoporosis drug salvianic acid A. The results showed that the bone-targeting liposomes treatment increased BMC and BV/TV compared to non-targeting liposomes.

Hyaluronic acid (HA) is a kind of naturally polysaccharide with excellent biocompatibility and plays a critical role in drug delivery [9, 10]. HA not only has an excellent biodegradability but also has high selective binding to CD44, which was increased in various cancer cells, including breast cancer, liver cancer, osteosarcoma and leukemia [11–13]. Owing to the excellent physiological characteristic and high chemical modification possibility, HA-CD44 interactions have been widely exploited for the treatment of malignant tumors.

In recently years, stimulate-responsive drug delivery attracted attention, including enzymes, acid, reactive oxygen and redox. Among them, the redox-activated therapy utilizing the disulfide bonds (-SS-) has been applied to realize the rapid release response of drug response to the intracellular redox potential of tumor cells [14]. Glutathione is a kind of small molecule which widely presented much higher concentrations inside the tumor cells (2–10 mM) than in the extracellular environment (2–20 μM), such as breast cancer, liver cancer and leukemia cells [15, 16]. This large discrepancy provides an ideal approach for designing a redox-sensitive drug delivery system for the treatment of malignant tumors.

**Figure 1.**
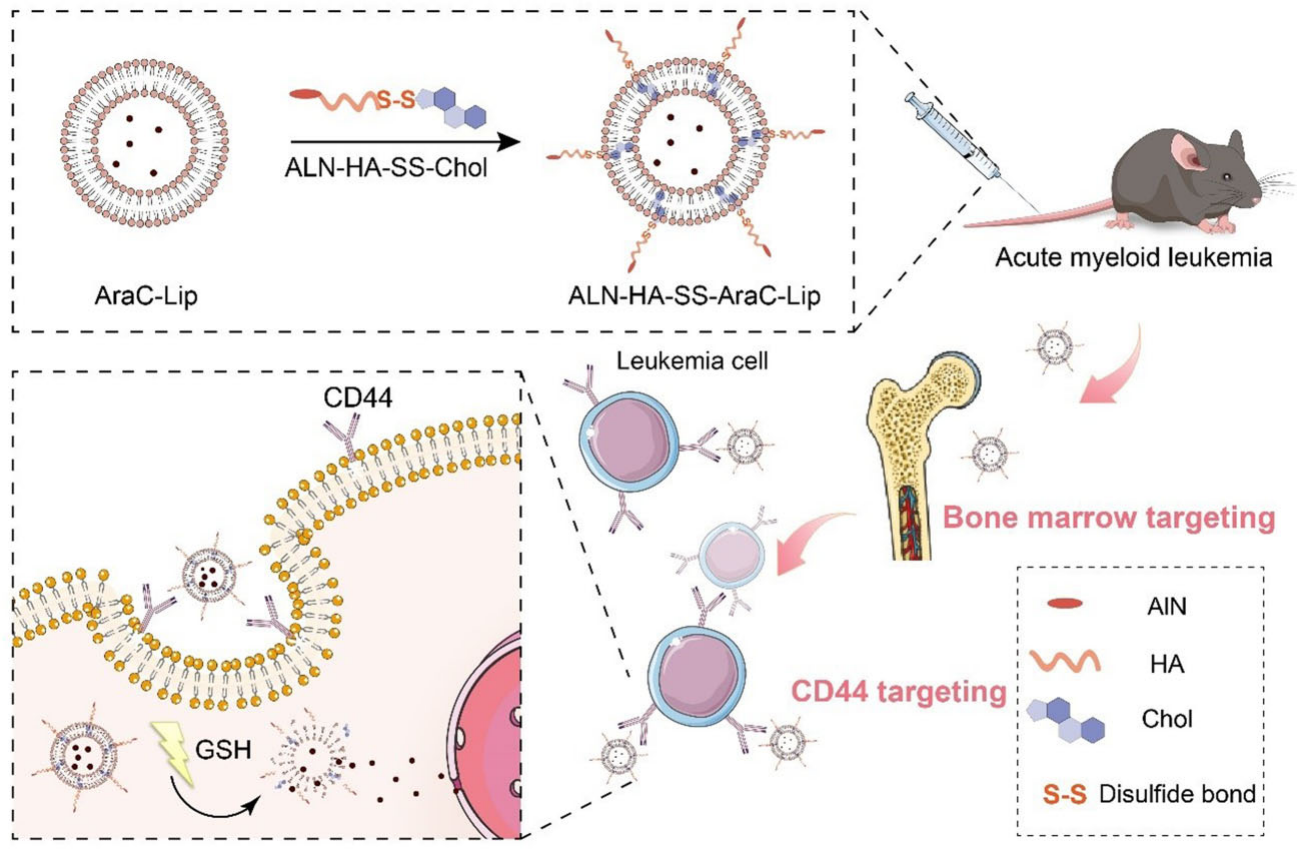
Illustration of bone-targeting liposomes system for AML therapy.

Liposomes are an ideal carrier for drug delivery, which are biocompatible and flexible with both hydrophilic and hydrophobic drugs inside the core and bilayer membrane, respectively [17]. Currently, appropriate modification could enhance the function of liposomes. For example, surface modification via the addition of a hydrophilic polymer, such as PEG and other hydrophilic materials, could reduce rapid elimination by phagocytic cells. In this study, we synthesized an amphiphilic polymer ALN-HA-SS-Chol. ALN and HA were linked with cholesterol by cystamine with -SS- bond and were used as targeting moieties of bone and AML cells (Fig. 1). These modified liposomes were utilized to deliver the antitumor drug, AraC. The no bone-targeting polymer liposomes or no redox sensitivity liposomes were synthesized for comparison.

## Materials and methods

### Materials

ALN and AraC were purchased from Shanghai Yuanye biology Co., Ltd (Shanghai, China). Low molecular weight hyaluronic acid (HA, MW: 5000–10 000 Da) was obtained from Shangdong Freda Biochem Co., Ltd (Jinan, China). ethylenediamine, pyrene1-ethyl-3 (3-dimethylaminopropyl) carbodiimide hydrochloride (EDCl) and 1-hydroxybenzotriazole monohydrate (HOBT), Cholesteryl chloroformate (Chol) and Cystamine dihydrochloride (Cys) were purchased from Macklin Reagent Co., Ltd.

For cell experiment, the mice leukemia C1498 cell line was obtained from Shanghai Zeye Biotechnology Co. D, L -dithiothreitol (DTT) was obtained from Aladdin Reagent Co., Ltd (Shanghai, China). Dulbecco's Modified Eagle's Medium, fetal bovine serum, Thiazolyl blue tetrazolium bromide (MTT), 4',6-diamidino-2-phenylindole (DAPI) and Indocyanine green (ICG) were purchased from KeyGEN BioTECH Co., Ltd (Nanjing, China).

For animal studies, female C57/BL6 mice (17–20 g) and male rats (220–240 g) were purchased from the Experiment Animal Center of Yangzhou University (Yangzhou, China), the animal experiments were performed according to the Ethical Committee of China Pharmaceutical University.

### Synthesis of the bone targeting modified HA derivatives

#### Synthesis of ALN-HA

ALN-HA was synthesized according to the previous reports with modification [18]. HA (400 mg, 1 mmol) was dissolved in 30 ml formamide and reacted with HOBT (0.68 g, 5.0 mmol) and EDCl (0.96 g, 5.0 mmol) for 2 h to activate the carboxyl moiety. ALN (136 mg, 0.5 mmol) was then dropwise into the above mixture and stirring for 24 h under the room temperature. The product was isolated by distilled water for 24 h [19]. Finally, the solution was lyophilized and the structure of ALN-HA was determined by ^1^H NMR.

#### Synthesis of reduction-sensitive conjugates

Cystamine dihydrochloride (2.25 g, 0.01 mol) and triethylamine (6.95 ml, 0.05 mol) were dissolved in 20 ml of dichloromethane (DCM). Meanwhile, 10 ml of DCM containing cholesteryl chloroformate (0.45 g, 0.001 mol) was added dropwise with constant pressure funnel over 4 h at 0°C [20]. The reaction was proceeded for further 10 h at the room temperature. The reaction device was carried out with the nitrogen atmosphere. The sediment was then eliminated by filtration and dried with anhydrous MgSO_4_. After dried in vacuum, the crude product was then purified with column chromatography by methanol: DCM of 1:20 (v/v) and the Chol-Cys was obtained.

#### Synthesis of reduction-insensitive conjugates

Chol-eda was obtained according to the previous reports. Triethylamine (1 mmol) and ethylenediamine (10 mmol) and were dissolved in 10 ml of DCM. The cholesteryl chloroformate (1 mmol) dissolved in DCM was added dropwise with an ice bath [21]. The above mixture solution was stirring for overnight at the room temperature under a nitrogen atmosphere, and the product was washed with distilled water for three times and dried with anhydrous MgSO_4_, at last, the desired product was purified with column chromatograph by methanol: DCM of 1:20 (v/v) and the Chol-Eda was obtained.

#### Synthesis and characterization of ALN-HA-SS-Chol

ALN-HA (0.5 mmol) was dissolved in formamide at 60°C, after complete dissolution, EDCl (1.5 mmol) and HOBT (1.5 mmol) were added into formamide and stirred for 1 h at the room temperature, the Chol-Cys (0.1 mmol dissolved in 30 ml DMF) was added into above mixture solution and stirred for further 48 h. the mixture solution was dialyzed (MWCO 3500 Da) against DMF and distilled water mixture solution for 24 h. The synthesis of HA-SS-Chol and HA-Chol were resemble to similar procedure. Besides, the content of ALN was evaluated by spectrophotometry, briefly, 0.5 ml of polymer was mixed with 0.2 ml of 6 mM FeCl_3_ and 1 ml of HClO_4_, and then the absorbance of the mixture at 300 nm was determined against the blank, the linear standard curve was prepared using ALN at the concentration range 0–5 mM.

### Preparation of liposomes

The liposomes were prepared according to the following steps, hydrogenated soybean phosphatidylcholine and cholesterol (2:1/mol:mol) were dissolved in 3 ml monomethyl ether, 2 ml of 10 mg/ml cytarabine solution was added and sonicated for 10 min, then 5 ml of PBS were added into bottle when the mixture solution turned to the milky state, after remove the solvent under vacuum and the cytarabine liposomes (AraC-Lip) were obtained. The liposomes were extruded through the polycarbonate membranes with the size of 0.45 μm, and the free AraC was removed by dialysis. Subsequently, the functionalized liposomes of formulation, HA-AraC-Lips, HA-SS-AraC-Lips and ALN-HA-SS-AraC-Lips were prepared by incubating the AraC-Lip at 60°C for 60 min with HA-Chol, HA-SS-Chol and ALN-HA-SS-Chol, respectively. Meanwhile, C6-loaded liposomes and ICG-loaded liposomes were prepared and decorated with the same way [21].

### Characterization of liposomes

#### Particle size, zeta potential and morphology

The particle size and zeta potential of the liposomes were measured by dynamic light scattering (DLS) using a Malvern Nano ZS (Malvern Instruments, UK), and the morphology and size distribution were observed and photographed by transmission electron microscopy (Hitachi HT7700 TEM, Japan).

#### Drug loading and encapsulation efficiency

The liposomes were digested in methanol containing 0.1% (w/v) Triton X-100 after ultracentrifugation. The digested mixture was centrifuged at 3000 r/min for 10 min, the drug content was analyzed by UV spectroscopy. The encapsulation efficiency (EE) (%) and drug loading (DL) (%) of liposomes were determined by the following equation:

$$\mathrm{EE}(\%)=\frac{\mathrm{AraC}\;\mathrm{weight}\;\mathrm{measured}\;\mathrm{in}\;\mathrm{liposomes}}{\mathrm{AraC}\;\mathrm{weight}\;\mathrm{added}\;\mathrm{in}\;\mathrm{liposomes}}\times \mathrm{100\%}$$

DL(%)=the weight of AraCThe weight of AraC and subsidiary material×100%.

#### Redox responsiveness of liposomes

To simulate the tumor microenvironment site of drug release, the AraC from liposome suspension and free AraC were evaluated using dialysis bags (MWCO 8 kDa), about 2 ml of liposome suspension was immersed in 30 ml of PBS (pH 7.4) containing 10 mM DTT and the samples were kept at 37°C with shaking, the content of AraC was determined by UV [22].

The redox sensitivity of liposomes was also evaluated by observing the size distribution, zeta potential and morphology after 4 h using DLS and TEM.

### *In vitro* cytotoxicity assay

The cytotoxicity of liposomes and free cytarabine was evaluated in murine leukemic cells by MTT assay. About 10^4^ cells were seeded in 96-well plates overnight, and the free AraC and liposomes were added to each well and incubated. Following 24 h of incubation, the cells were incubated with 20 μl MTT solution (5 mg/ml) for another 4 h. The supernatant was removed and the formazan crystals were dissolved in DMSO. The absorbance value was measured at 570 nm using an Elx800 microplate reader. Cells without liposomes or free cytarabine treatment were used as a negative control, cell viability was calculated as (OD test group/OD control group) × 100% [23, 24].

### HAP binding test

Bone affinity of free cytarabine and liposomes was evaluated by HAP binding test. In brief, liposomes and free AraC were dissolved in PBS (pH = 7.4, 1 mg/ml). The above PBS was incubated with HAP powder (100 mg) to mimic physiological conditions of bone tissue in body, the samples were centrifuged at 3000 r/min for 3 min at predetermined time points. The binding efficiency was evaluated by calculating the fluorescent intensity loss of the corresponding supernatant [25, 26].

### *In vitro* cellular uptake of liposomes

Cellular uptake of liposomes by C1498 cells was compared using Confocal laser scanning microscopy (CLSM). C1498 cells were incubated in glass bottom cells at the density of 10^4^ cells/well overnight. C6-loaded liposomes were diluted with medium to the concentration of 10 μM and then added to plates. The cells were then cultured for 4 h and the medium were discarded, the cells were rinsed three times with PBS and fixed with paraformaldehyde at the room temperature for 30 min. The cells were then washed for three times with PBS, and the cell stained by 100 μl DAPI for 30 min [27]. After washing with PBS for three times, the images were obtained with the CLSM (LSM800, Carl Zeiss, Germany).

In another experiment, the cells were seeded in the six-well plates overnight, and the cells were incubated with above formulation, followed by washed and suspended with PBS, the fluorescence of C6 was evaluated using the flow cytometer (BD, USA) [28].

### Biodistribution studies

In this study, ICG-loaded liposomes were prepared according to the previous literature. The mice were treated with free ICG, ICG-Lips, HA-SS-ICG-Lips and ALN-HA-SS-ICG-Lips at the concentration of 5 mg/kg by tail vein. After 12 h, the mice were euthanized, the main organ and the bone were removed and washed by PBS. The fluorescence in organs and bone was analyzed by IVIS-Spectrum device (PerkinElmer, American) [29, 30].

### *In vivo* efficacy study

C57/BL6 mice were randomly divided into six groups. C1498 cells (5*10^6^) were injected via the tail vein, 7 days later, each group of mice was injected with saline, free AraC, AraC-Lip, HA-SS-AraC-Lip and ALN-HA-SS-AraC-Lip at the dose of 5 mg/kg, and the mice were treated for six times at 3-day intervals. After the treatment, three mice from each group were sacrificed, the spleen of mice were extracted and weighed, the cell morphology of bone marrow and peripheral blood were stained with Wright-Giemsa. The other five mice from each group were used to recorded the survival rates [31].

### Pharmacokinetic study

The pharmacokinetic assays were performed using SD rats, the rats were divided into three groups and injected by free AraC, AraC-Lip and ALN-HA-SS-AraC-Lip (5 mg/kg) were injected into the tail vein. Blood samples (0.2 ml) were collected from the retro-orbital plexus and placed into the heparinized tube, the samples were centrifuged (14 000×g, 15 min) and 50 μl of glacial acetic acid and 0.8 ml acetonitrile were added to the plasma, followed by vortex-mixed and centrifuged at 1000×g for another 10 min at 4°C, the supernatants were dryness under the nitrogen flux at 42°C and then filtered through a 0.22 μm pore size syringe filter [32]. The samples were analyzed using an HPLC system. Drug and Statistics (DAS, Version2.0) were used to calculate the pharmacokinetic parameters.

### Statistical analysis

Data were showed as mean ± SD. Statistical significance was evaluated with Student’s *t*-test using GraphPad Prism 8 software. *P* < 0.05 was considered statistically significant.

## Results

### Design and synthesis of HA derivatives

**Figure 2.**
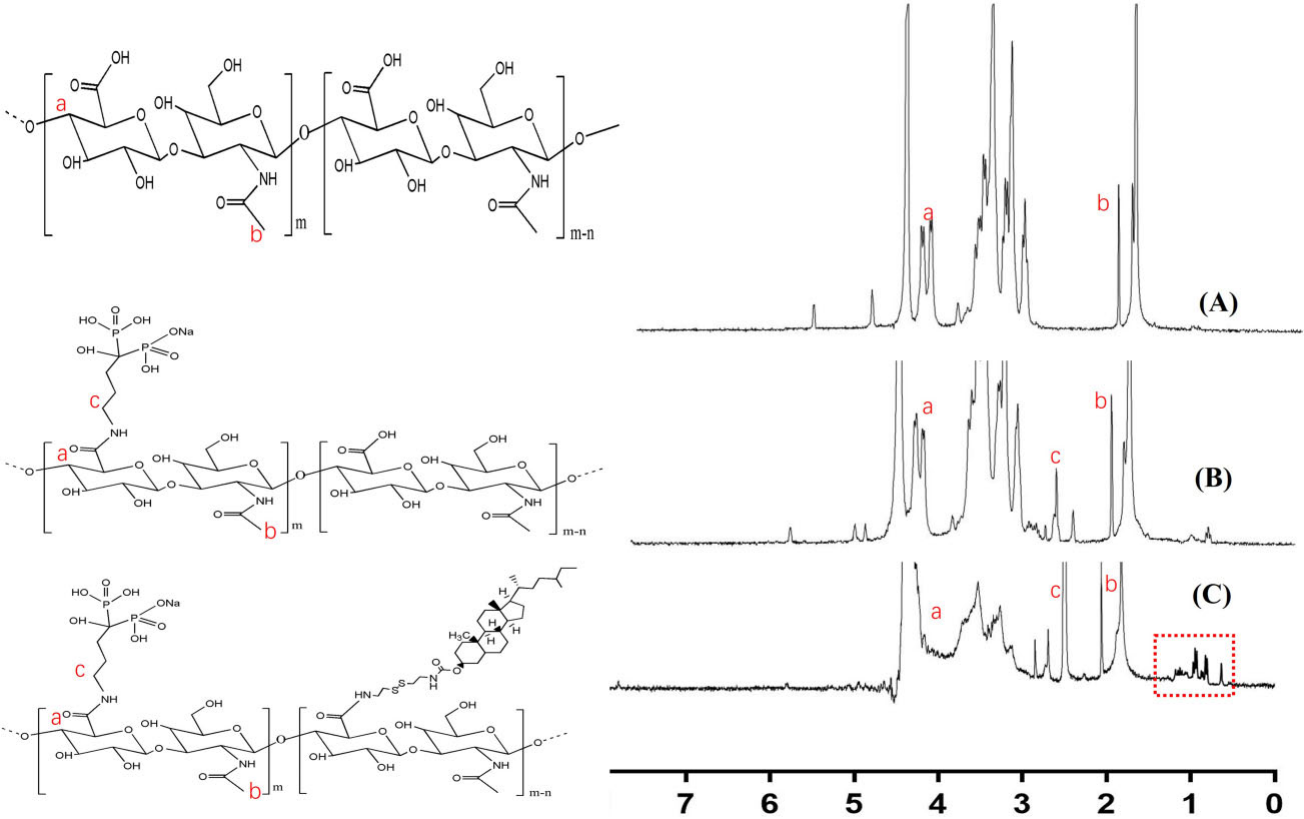
^1^H NMR Spectra of HA, ALN-HA and ALN-HA-SS-Chol. The solvents were D_2_O, D_2_O and D_2_O/DMSO-d6(1/3, v/v), respectively.

The synthesis route and ^1^H NMR of HA derivatives were shown in Supplementary Fig. S1 and Fig. 2, respectively. The peaks at 1.9 and 2.9–3.0 ppm were contributed to the methyl protons of the acetyl amide group and rings of HA [33], the characteristic peak of ALN was shown in 2.85 ppm indicated that the ALN had successfully graft on the HA, the degree of substitution of ALN in ALN-HA-SS-Chol was 9.3%. Besides, the peaks at around 1 ppm were attributed to the Chol in Fig. 2C, indicating that the Chol was successfully grafted on the ALN-HA. Besides, intermediate product of eda-chol and cys-chol were also vitrificated by mass spectrometry in Supplementary Figs S2 and S3.

### Physicochemical characteristics of liposomes

The liposomes formulation exhibited the average size of 90–120 nm (Table 1) and all HA-hmodified liposomes showed negative zeta potentials, which attributed to the –COO- group of HA. Besides, all groups of liposomes showed the EE of around 60% and the DL of over 7%, which exhibited the good performance in encapsulating AraC.

**Table 1.** Characteristics of different AraC-loaded liposomes (*n *=* *3)

Liposomes	Size (nm)	Zeta (mV)	EE (%)	DL (%, w/w)	PDI
AraC-Lip	93.5 ± 2.1	1.2 ± 0.02	58.6 ± 0.8	7.3 ± 0.14	0.18 ± 0.01
HA-AraC-Lip	108.6 ± 5.3	−19.8 ± 1.7	60.1 ± 1.1	7.6 ± 0.13	0.20 ± 0.01
HA-SS-AraC-Lip	115.3 ± 6.1	−21.5 ± 0.8	59.2 ± 0.9	7.9 ± 0.17	0.20 ± 0.01
ALN-HA-SS-AraC-Lip	117.5 ± 6.0	−20.6 ± 1.1	58.3 ± 0.8	7.4 ± 0.16	0.19 ± 0.01

### Characterization of reduction-sensitive of the liposomes

**Figure 3.**
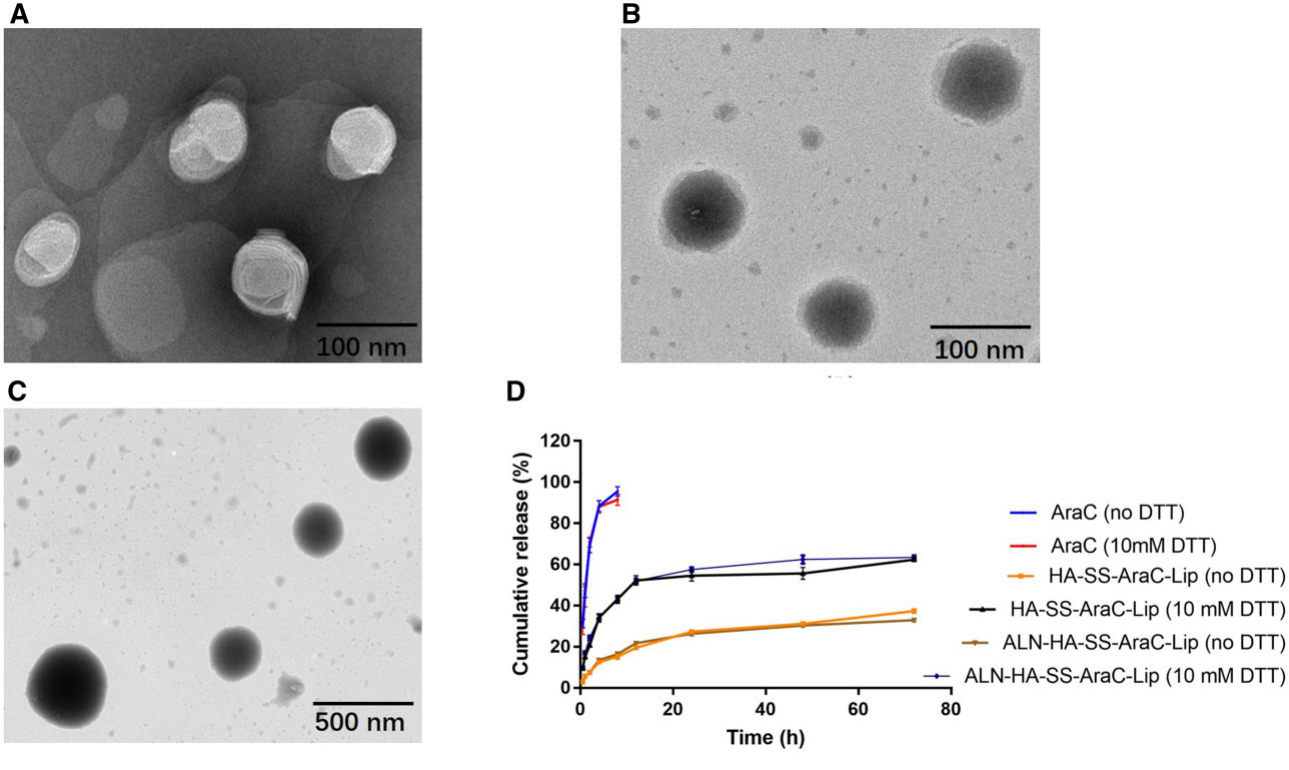
(**A**) TEM Images of AraC-Lip, (**B**) ALN-HA-SS-AraC-Lip in PBS and (**C**) ALN-HA-SS-AraC-Lip in 10 mM DTT. (**D**) Release profiles of AraC from liposomes in different environment. 37°C (*n *=* *3).

The AraC-Lip and ALN-HA-SS-AraC-Lip were showed generally spherical in the TEM images with the diameters of about 100 nm, which was similar as the DLS (Fig. 3A). Although the liposomes remained as the spheres in PBS containing 10 mM DTT after 4 h, the TEM showed that the liposome features had vanished, and the size distribution also indicated that the liposomes were expanded and broken after incubated with DTT (Supplementary Fig. S5). The results showed the disulfide-decorated liposomes not only possessed the stability with the normal physiological conditions but also expanded under the reductive environment which simulated tumor intracellular environment (Fig. 3B and C). AraC release from liposomes in the PBS or 10 mM DTT was evaluated to simulate the physiological conditions and the tumor environments, respectively. As shown in Fig. 3D, nearly 100% free AraC was released out of the dialysis bag within 8 h, however, slow drug release from all group of liposomes in the PBS, for example, only about 20% of AraC were released in 72 h, at the condition of 10 mM DTT, the HA-SS-AraC-Lips and ALN-HA-SS-AraC-Lips groups released about 60% AraC in 24 h. On the contrary, AraC release from AraC-Lip and HA-AraC-Lips was not related to the concentration of DTT (Supplementary Fig. S6). The results indicated that the liposomes with -SS- structure could achieve intracellular drug release in tumor microenvironment.

### *In vitro* cell cytotoxicity study

**Figure 4.**
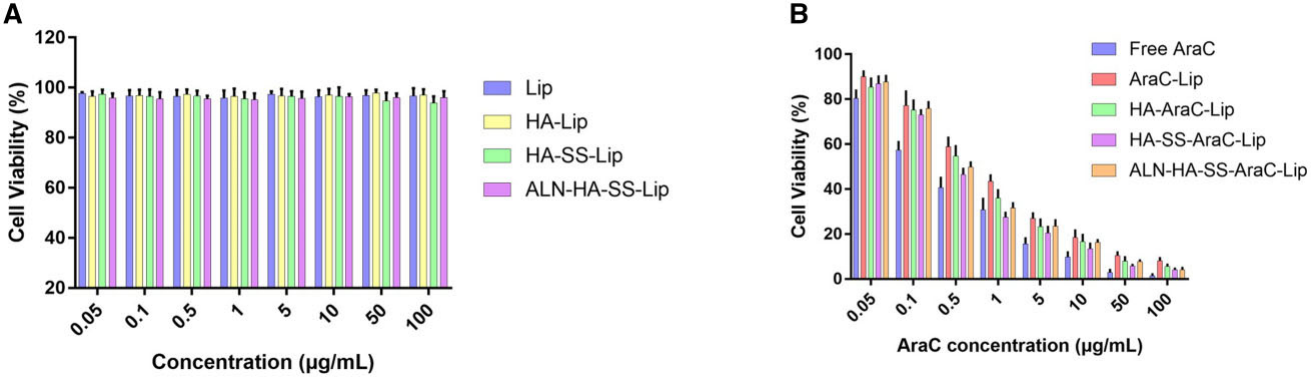
Cytotoxicity assays of free AraC and AraC loaded liposomes (**B**) and blank liposomes (**A**) on C1498 cells (*n *=* *6).

*In vitro* cell cytotoxicity of different formulation was estimated by MTT experiment in C1498 cells. As shown in Fig. 4B, MTT assays showed that all groups of blank liposomes had no toxicity at a high concentration on C1498 cells, which illustrated that the polymers and liposomes were biocompatible and safe. The antitumor activity of free AraC and AraC-loaded liposomes on C1498 cells showed a dose-dependent manner. The IC50 values of free AraC, AraC-Lips, HA-AraC-Lips, HA-SS-AraC-Lips and ALN-HA-SS-AraC-Lips were 0.42, 0.93, 0.72, 0.56 and 0.60 μg/ml after incubation for 24 h, respectively. Similar with other studies, free AraC possessed the most cytotoxic effect than the liposomes groups due to the immediate access to cell as the small molecule. The liposomes modified with HA produced a small IC50 than AraC-Lip group, which is because of the HA is beneficial to the uptake of liposomes by C1498 cells. Furthermore, the polymer modified with disulfide bond had lower IC50 value than HA-AraC-Lips (0.56 and 0.60 vs 0.72 μg/ml). The similar result was also reported that disulfide bonds are beneficial for improving the antitumor activity.

### *In vitro* HAP binding study

**Figure 5.**
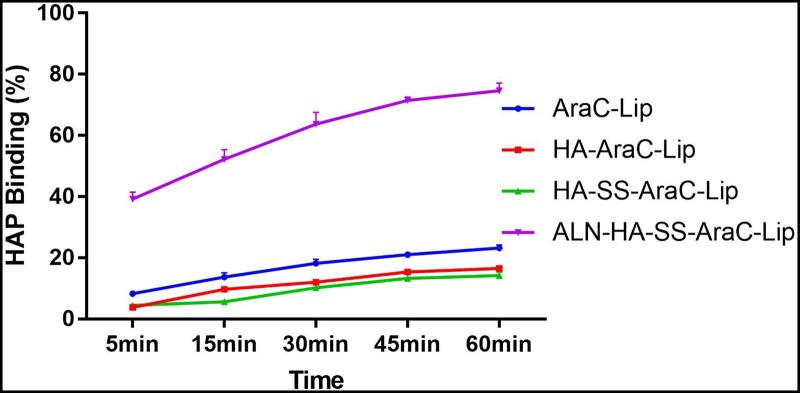
Binding ability of different modified liposomes to HAP (*n *=* *3).

The ability of each group of liposomes binding to HA was shown in Fig. 5, the efficiency of liposomes with no ALN binding to HA was <20% after 1 h. However, the ALN-decorated liposomes exhibited a high binding affinity, over 60% and ∼80% of ALN-HA-SS-AraC-Lips binding to HA with stirring after 30 and 60 min, respectively. According to the above results, the ALN-HA-SS-AraC-Lips group showed excellent HAP affinity *in vitro*.

### Cellular uptake

**Figure 6.**
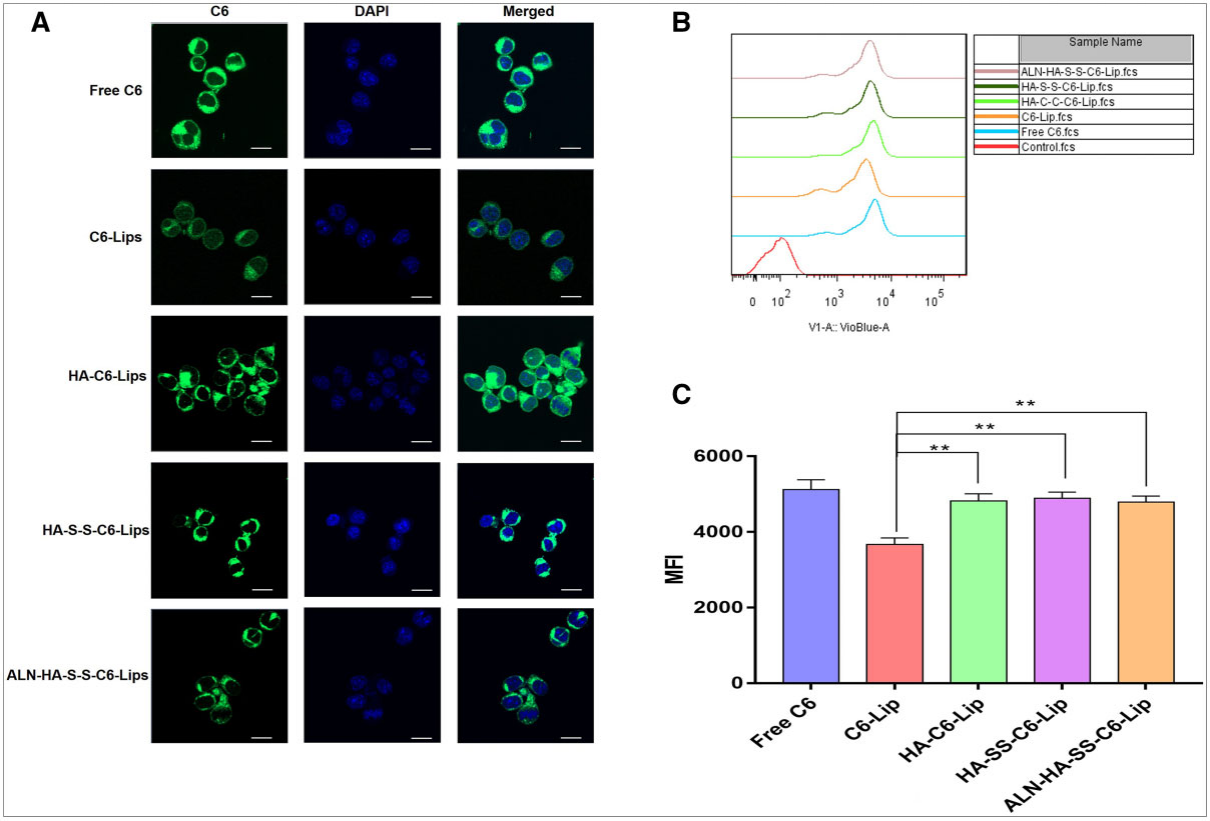
Cellular uptake of different formulation in C1498 cell. (**A**) Confocal microscopy images of C1498 cell incubated with free C6 and C6-loaded liposomes for 2 h at 37°C (scale bar = 10 μm). (**B**) Flow cytometry histograms and (**C**) cytotoxicity assays for C6 and C6-loaded liposomes on C1498 cells after 2 h incubation; the fluorescence intensity was measured for three times.

C6 is a fluorescent dye widely used in cellular uptake experiment as a substitute for drugs. In this study, C6-loaded liposomes formulations were prepared and used in C1498 cells for uptake experiments, as shown in Fig. 6A, CLSM was applied to evaluate the cellular uptake of C6, after incubation for 2 h, C1498 cells treated with C6-Lips exhibited the weaker fluorescence intensity than the free C6 group, however, after decorated with the HA, the fluorescence intensity of liposomes increased compared with the C6-Lips group, indicating that increased uptake of liposomes modified with HA of C6 than incubation of C6-Lips.

Flow cytometry was applied to evaluated the cellular uptake of free C6 and liposomes. After incubation for 2 h, it showed a distinct different performance between free C6 and liposome formulations in the cellular uptake (Fig. 6B). Quantitative analysis results of the flow cytometry exhibited the similar trend as the CLSM, and the results of cellular uptake results seemed to be related to the MTT assays.

### *In vivo* biodistribution of liposomes

**Figure 7.**
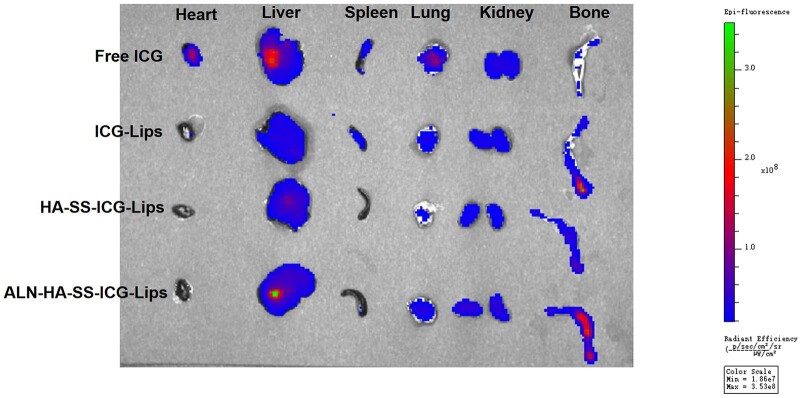
Tissue distribution of ICG detected by living images system at 12 h after free ICG and ICG-loaded liposomes were intravenously to C57/BL6 mice.

The *in vivo* biodistribution of free ICG and liposome formulation was shown in Fig. 7. After 12 h, free ICG was mainly distributed in the main organ including heart, liver, spleen, lung and kidney, however, ICG-loaded liposomes displayed an increasing accumulation in bone. Compared with ICG-Lips and HA-SS-ICG-Lips, ALN-HA-SS-ICG-Lips showed the strongest fluorescence signal in bone, indicating that ALN is beneficial to the aggregation of the liposomes in the bone site.

### *In vivo* antitumor activity

**Figure 8.**
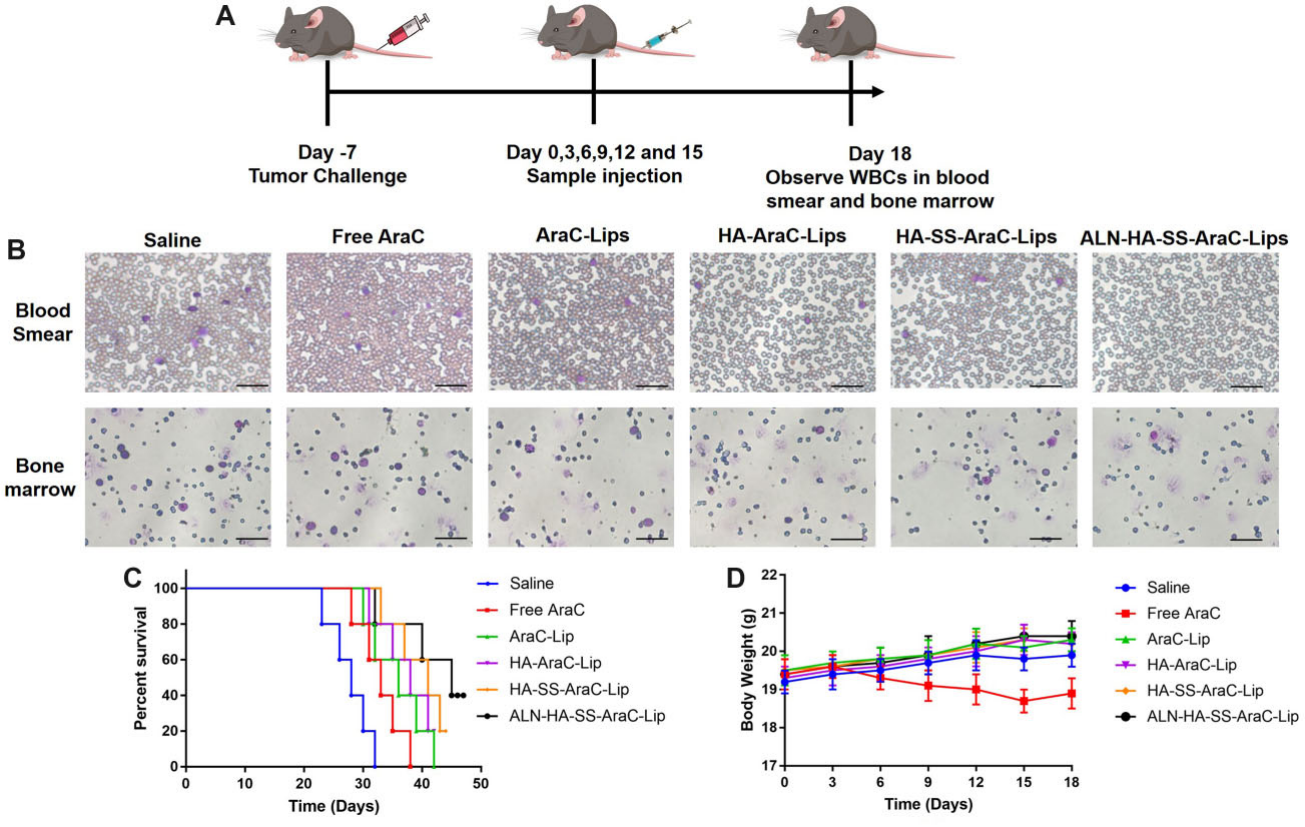
Antitumor activity of free AraC and AraC-loaded liposomes. (**A**) Treatment schedules for the indicated treatment. (**B**) Wright-Giemsa staining of blood smear and bone marrow in each group, purple stoods for WBCs (scale bar = 50 μm). (**C**) The survival curve of leukemia-bearing mice calculated by Kaplan–Meier estimate (*n *=* *5). (**D**) Mean weight of mice after the treatment (*n *=* *3).

To validate the antitumor efficacy, the C1498 tumor-bearing mice were treated as shown in Fig. 8A, *in vivo* antitumor activity was confirmed by the stained blood stream and bone marrow by Wright-Giemsa, the white blood cells (WBCs) decreased obviously after the treatment. As shown in Fig. 8B, abundant WBCs were shown in blood stream and bone marrow in saline group (red arrow), indicating the abnormal proliferation of WBCs, the number of WBCs decreased after the treatment, which attributed to the antitumor efficacy of AraC, compared to the AraC group, the liposomes showed the better antitumor efficacy, which may be related to the long circulation of liposomes. Besides, the treatment of HA-SS-AraC-Lips group could further eliminate the WBCs in blood and bone marrow for CD44 targeting and redox-responsive. Among them, ALN-HA-SS-AraC-Lips group could eliminate the residual leukemia cells in blood stream and bone marrow to the greatest extent, the body weight of mice was shown in Fig. 8D, free AraC exhibited the weight loss during the treatment, on the contrary, the liposomes group of mice showed the weight sightly weight gain. Remarkably, Kaplan–Meier survival curve showed that the C1498 tumor-bearing C57/BL6 mice treated with saline all dead within 32 days, the mice treated with pharmaceutical formulation were prolonged compared with saline group, among them, ALN-HA-SS-AraC-Lips group exhibited the best therapeutic effect. Forty percent of mice survived over 45 days, which significantly higher than other groups (Fig. 8C), and the degree of the anti-tumor efficacy ranked as the following order: ALN-HA-SS-AraC-Lips > HA-SS-AraC-Lips > HA-AraC-Lips > AraC-Lips > AraC. Besides, spleen morphology and spleen weight were also evaluated during the treatment, the ALN-HA-SS-AraC-Lips group also reduced the spleen weight which induced by the tumor cell infiltration (Supplementary Fig. S4).

### Pharmacokinetic study

**Figure 9.**
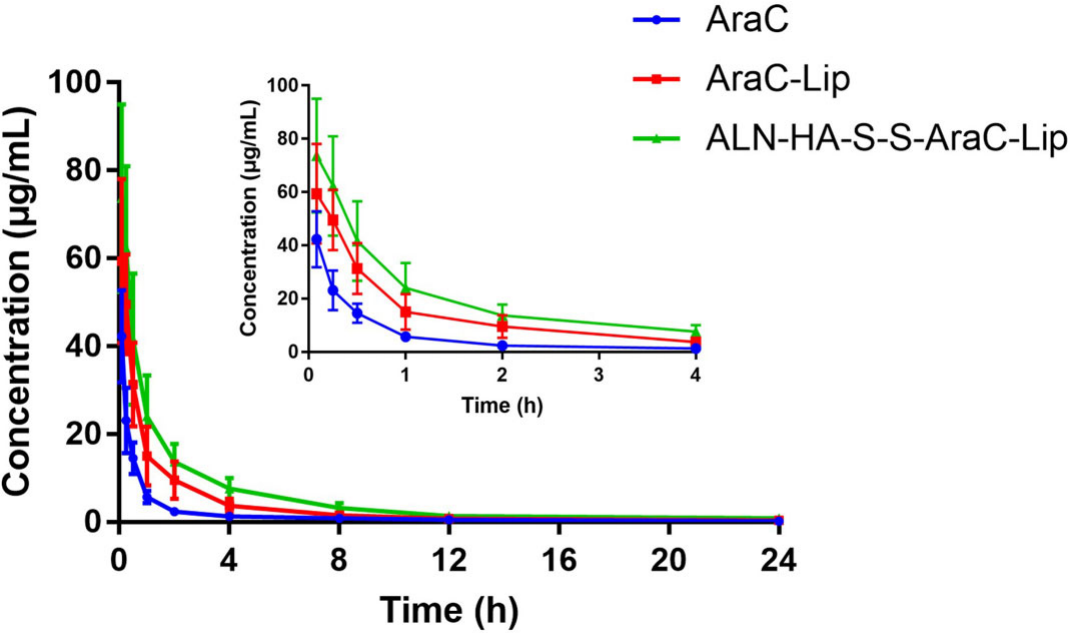
Plasma concentrations of AraC in SD rats after the intravenous administration of AraC, AraC-Lip and ALN-HA-SS-AraC-Lips with the AraC dose of 5 mg/kg (*n *=* *3).

To evaluate the *in vivo* AraC concentration, the pharmacokinetic of intravenous administration of different formulations was evaluated. The AraC concentration-time curve was shown in Fig. 9, the free AraC showed the rapidly disappearance from the circulation, showing the mean residence time (MRT_*0-t*_) of 1.27 h, on the contrary, AraC-Lips and ALN-HA-SS-AraC-Lips exhibited a longer residence time than the free AraC (Table. 2), on the one hand, the liposomes could significantly prolong the circulation time of the drugs, on the other hand, the HA derivative shell could prevent the absorption of plasma proteins and the uptake of RES, preventing AraC from the rapid clearance [34]. In addition, the area under curve (AUC μg/ml/h) of AraC-Lips and ALN-HA-SS-AraC-Lips also increased obviously compared to free AraC group (*P *<* *0.05), and the CL value also decreased to 0.14 ± 0.05 in AraC-Lips and 0.09 ± 0.04 in ALN-HA-SS-AraC-Lips compared to 0.52 ± 0.11 in free AraC group, the results showed that AraC-Lips and ALN-HA-SS-AraC-Lips could improve the circulation time and plasma concentration of AraC.

**Table 2.** Pharmacokinetics parameters of AraC and AraC-loaded liposomes after intravenous administration (*n *=* *3)

Samples	*C*_max_ (μg/ml)	AUC*_0-t_* (μg/ml/h)	MRT_*0-t*_ (h)	CL (l/h/kg)
AraC	42.1 ± 8.9	14.31 ± 3.42	1.27 ± 0.19	0.52 ± 0.11
AraC-Lip	59.7 ± 13.7	37.62 ± 8.65^*^	2.52 ± 0.34^*^	0.14 ± 0.05^*^
ALN-HA-SS-AraC-Lips	76.3 ± 21.3^*^	46.31 ± 9.12^**^	2.94 ± 0.41^*^	0.09 ± 0.04^**^

* *P *<* *0.05 vs free AraC

** *P *<* *0.01 vs free AraC

## Discussion

Bone marrow is the key hematopoietic organ, which is related to multiple malignant diseases including multiple myeloma, bone metastasis from solid tumor and AML [35]. These malignancies affect the normal internal environment balance and reshape the bone marrow microenvironment. The treatment is limited due to the inevitable deterioration and special physiological structure characteristics, the bone marrow targeted drug delivery systems are intended for the treatment of bone marrow tumor, which can suppress the growth of tumors in the bone marrow and reduce the side effects on healthy tissue.

As with the development of various smart drug delivery system, more and more good job has made significative attempts to combat the various tumor models. For example, Wu’s group synthesized Chol-SS-PEG by PEG with cholesterol by a bio-reducible disulfide linker to prepared the liposomes to deliver the DOX, exhibiting excellent antitumor activity in MG63 osteosarcoma cancer than normal liposomes group [36]. Also, Ronit’s group developed PEG conjugated aspartic acid modified liposomes as the bone-targeting carrier for paclitaxel, the *in vitro* and *in vivo* experiment showed the excellent bone affinity ability of bone targeting liposomes, and the liposomes exhibited ideal therapeutic efficacy in animal model [37]. In this research, we evaluated the therapeutic effects of liposomes on AML model. The bone and CD44 dual targeting liposomes with redox-sensitive were prepared by inserting amphiphilic polymer into liposomes. The multi-function liposomes were prepared on the following steps. First, ALN was modified onto the carboxyl of HA, and the cholesterol was grafted onto the HA by the cysteamine. The mass spectrometry and the ^1^H-NMR showed the ALN-HA-SS-Chol were successful synthesized (Fig. 2), meanwhile, HA-SS-Chol and HA-Chol were also synthesized for comparison. As presented in the TEM images, the AraC-Lip and ALN-HA-SS-AraC-Lip possessed a spherical morphology with the mean particle size of around 100 and 120 nm, respectively (Table 1 and Fig. 3A–C). After incubation in 10 mM DTT, the ALN-HA-SS-Lip remained as spheres, but the lipid bilayer structure disappeared, which induced by the disulfide bond of ALN-HA-SS-Chol [38]. The *in vitro* release also showed that ALN-HA-SS-Lip could release AraC faster at the environment of 10 mM DTT (Fig. 3D). HA has been widely studied as drug delivery system for decorated nanoparticles in drug chemotherapy, which can specifically recognize cell-specific surface markers such as CD44 receptors over-expressed by tumor cells [18, 39]. To investigate the affinity ability of liposomes modified with HA, we used flow cytometry and CLSM for the cellular uptake study of liposomes in C1498 cells. The results showed that the fluorescence signals of C1498 cells line treated with C6-Lips decreased than C6 solution, which related to phospholipid bilayer of liposomes inhibited the cellular uptake due to the shied effect. However, the HA modified liposomes increased the fluorescence signals compared with the C6-Lips. Meanwhile, modified with ALN did not affect the CD44-targeting property (Fig. 4A). Flow cytometry showed the same results as the CLSM (Fig. 4B and C), the results of cellular uptake results seem to be correlated with the MTT assay results, free AraC induced the strongest cytotoxic effect compared with the liposomal formulations, which might due to the immediate access to the cells as a small molecule, after decorated with HA, the IC50 value decreased compared with AraC-Lips group (Fig. 4A). CD44-mediated liposomes entry C1498 cells and the cytotoxic drug AraC could be released from liposomes and exerted a better antitumor therapeutic effect compared with free AraC and AraC-Lips groups. Besides, the ALN-HA-S-S-AraC-Lips also exhibited the longer circulation time compared to the AraC-Lips group, which may be attributed to the HA shell of the liposomes, the hydrophilicity of HA could prevent the uptake of reticuloendothelial system and adsorption of plasma protein.

To observe whether the bone-targeting liposomes could effectively deliver the drug to the bone site and achieve high therapeutic efficacy, the free ICG and ICG-loaded liposomes were used to evaluate. After the free ICG and ICG-loaded liposomes injection, the free ICG was distributed to heart, liver, lung and kidney tissue, and the fluorescence distribution to the bone was very low. Meanwhile, in the ICG-loaded liposomes groups, the fluorescence signal in bone of ICG was higher than free ICG group, among them, the bone-targeting liposomes group showed the strongest fluorescence signal (Fig. 7), besides, *in vitro* affinity experiment also proved that the liposomes modified with ALN were beneficial to the accumulation of HAP (Fig. 5). The reason might be that liposomes are beneficial to the long circulation of the drugs and ALN mediated bone targeting efficiency. The high accumulation of drugs in the bone site can contribute to the antitumor efficiency in AML mice model. To observe the antitumor efficiency of the functionalized liposomes in AML mice model, we observed the count of peripheral WBCs in the blood smear and bone marrow. The number of WBCs in blood smear and bone marrow is an important standard in AML model, compared with other groups, the bone-targeting liposomes group could eliminate the leukemia cells in the blood smear and bone marrow to the greatest extent (Fig. 8). Spleen is an important immune organ, splenomegaly is almost constant in ALM model [40], therefore, spleen is also a key indicator to assess the efficiency of the drug formulations, splenomegaly was serious in the model group and even reached about 350 mg (Supplementary Fig. S7) which similar with the previous reports [41]. After the treatment, all groups of spleen weight decreased, among them, the bone-targeting group showed the best efficiency, that is, the heavier the spleen weight, the stronger the AML degree. The mean spleen weight of the ALN-HA-SS-AraC-Lips group was the smallest compared with the other groups, indicating that the bone-targeting group has the strongest antitumor ability. At last, survival time was also recorded during the treatment, the saline group began to die from 23 days and all mice die in 32 days (initial mice number was 5), after the treatment, the mice could extend the overall survival time, among them, the bone-targeting redox liposomes showed the strongest antitumor efficiency and 40% of mice still alive after 45 days (Fig. 8A).

Taken together, *in vivo* experiments indicated that the redox sensitivity liposomes with bone and CD44 dual targeting enabled a specific and efficient delivery of the AraC to the leukemia cells, which could kill the leukemia cells in mice.

## Conclusion

In this study, AraC-loaded liposomes with ALN and HA as dual-targeting ligands on the surface were successfully developed by postinsertion manner, and the liposomes decorated with ALN-HA on the surface via a bio-reducible linker (-SS-) could achieve bone site and leukemia cells targeting in turn, our experiment revealed that the formulation had the strong affinity to HAP *in vitro* and accumulation capacities of bone-targeting *in vivo*. When tested in the AML mice model, the bone-targeting liposomes were found to significantly decreased the WBCs and prolong the survival time.

## Supplementary data

Supplementary data are available at *REGBIO* online.

## Funding

This work was supported by the Chuzhou University Scientific Research Fund (2020qd50, 2022XJYB10), the Key Research and Development Program of Anhui Province (202104b11020010) and the Students' Innovation and Entrepreneurship Training Program of Anhui Province (S202110377140).

*Conflicts of interest statement.* None declared.

## Supplementary Material

rbac058_Supplementary_Data
