# Supplementary material for: Cytarabine delivered by CD44 and bone targeting redox-sensitive liposomes for treatment of acute myelogenous leukemia
Source: Regen Biomater. 2022 Aug 24;9:rbac058. doi: 10.1093/rb/rbac058 (PMC9469920; doi:10.1093/rb/rbac058)
Supplement: rbac058_Supplementary_Data [file rbac058_supplementary_data.docx]

**Supplementary data**

**Cytarabine delivered by bone and CD44 dual targeting redox sensitive liposomes for treatment of acute myelogenous leukemia**

Hao Wu^1^ ^†^, Yuan Gao ^2^ ^†^, Jia Ma^3^ ^†^, Maosong Hu^1^, Jing Xia^1^, Shuting Bao^1^, Yuxi Liu^1*^, Kai Feng^3*^

*1.College of Materials & Chemical Engineering, Chuzhou University, Chuzhou 239000, China;*

*2. Department of Oncology, Beijing Shunyi Hospital, NO.3 Guangming South Street, Shunyi District, Beijing 101300, China;*

*3.Department of Neurology, Beijing Shunyi Hospital, NO.3 Guangming South Street, Shunyi District, Beijing 101300, China;*


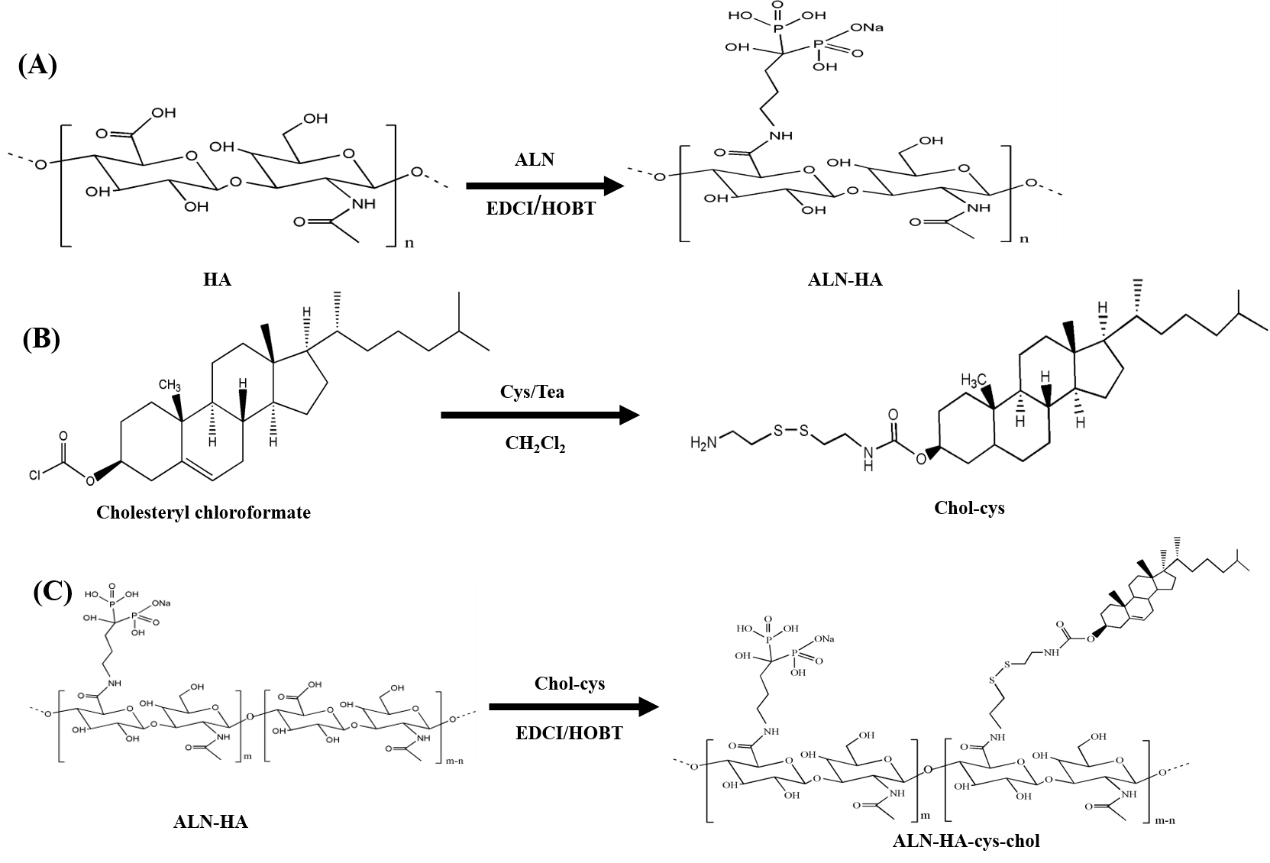


**Fig. S1. Synthesis diagram of ALN-HA-SS-Chol**

**Fig. S2.** MS of Chol-Eda in CDCl_3_

**Fig. S3.** MS of Chol-Cys in CDCl_3_


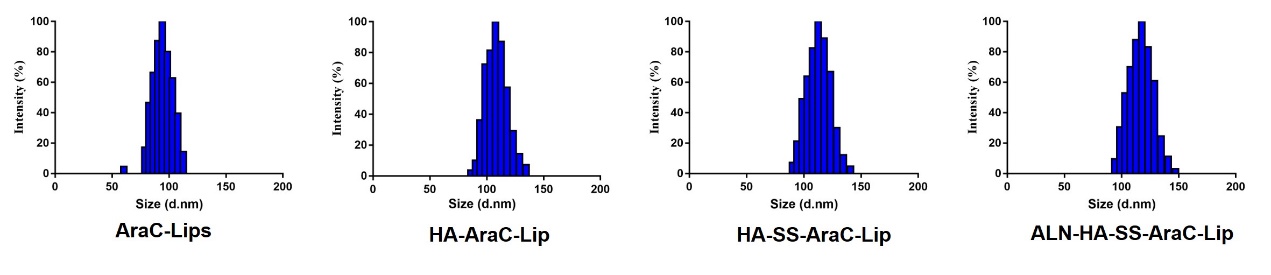


**Fig.S4.** Size distribution of different group of liposomes


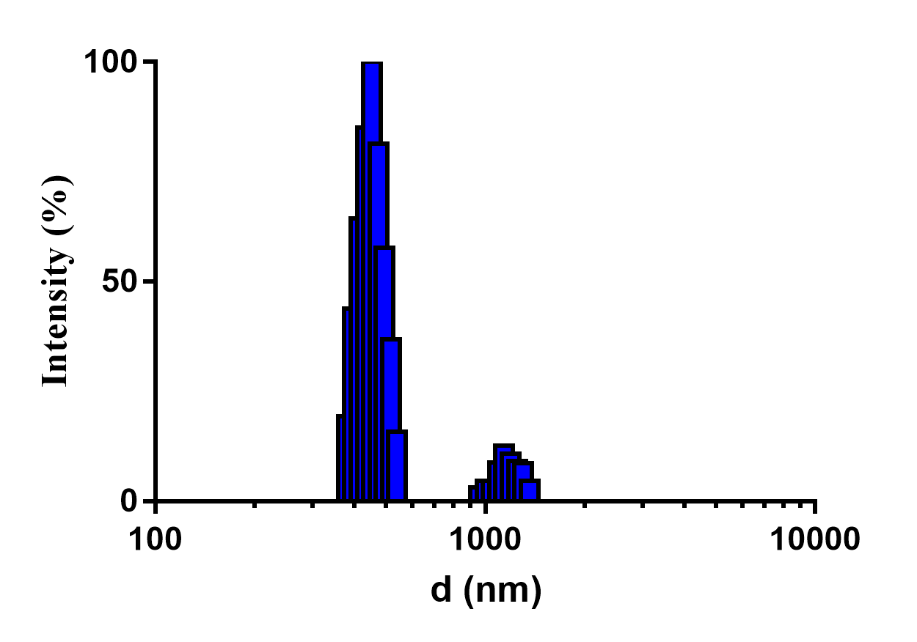


**Fig.S5** Size distribution of ALN-SS-HA-AraC-Lips after incubation with 10 mM DTT.


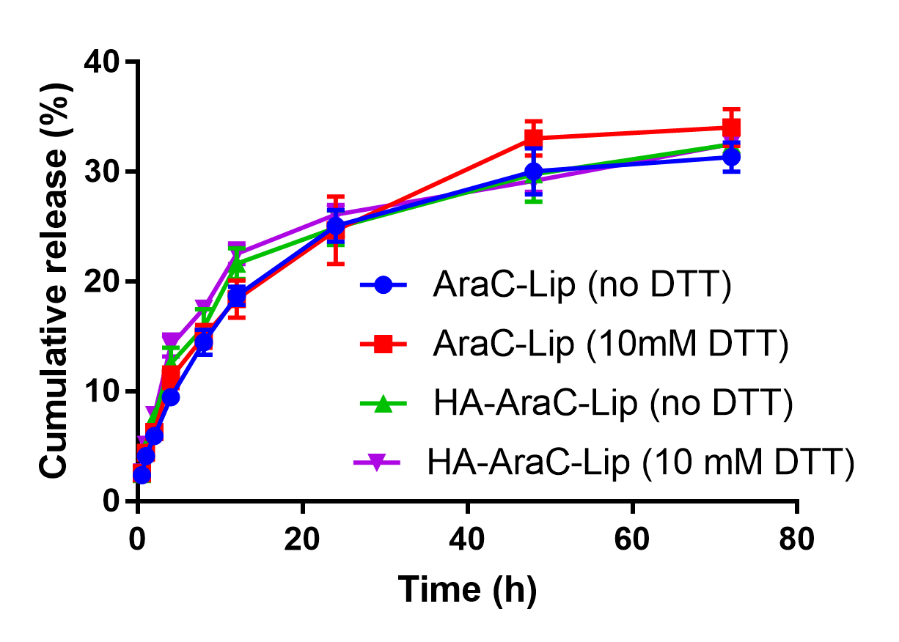


**Fig.S6**. Release profiles of AraC from liposomes in different environment. 37 °C (*n* = 3)


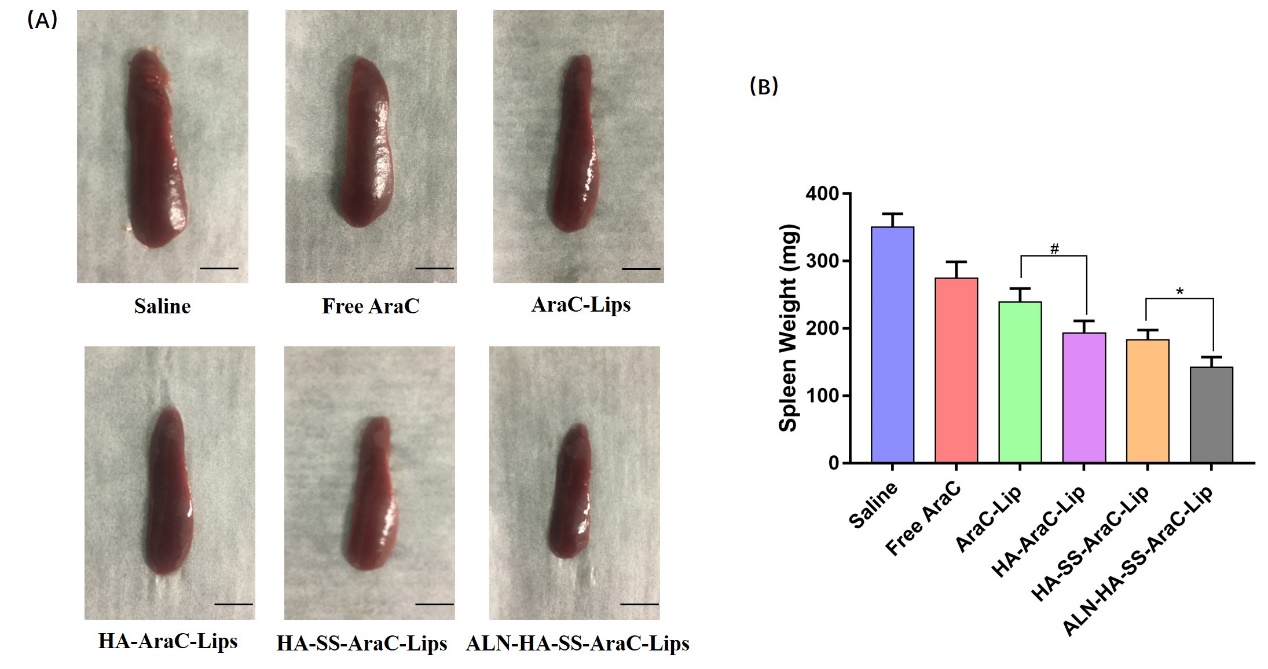


**Fig. S7.** Spleen morphology and spleen weight of AML model mice after treatment (Scale bar: 1cm)
